# Supplementary material for: Effectiveness and Safety of Ayurvedic Medicines in Type 2 Diabetes Mellitus Management: A Systematic Review and Meta-Analysis
Source: Front Pharmacol. 2022 Jun 8;13:821810. doi: 10.3389/fphar.2022.821810 (PMC9213670; doi:10.3389/fphar.2022.821810)
Supplement: Supplementary file 1 [file DataSheet1.zip › Supplementary Material/Forest and Funnel Plots/Trigonella foenum-graecum/TC.pdf]

Trigonella foenum-graecum - TC

Study	Intervention N	Intervention Mean	Intervention SD	Comparator N	Comparator Mean	Comparator SD	Mean Diff. with 95% CI	Weight (%)
Gupta 2001	12	186.70	38.00	13	209.30	38.30	-22.60 [-52.54, 7.34]	12.42
Rafraf 2014	44	93.90	21.14	44	104.00	20.94	-10.10 [-18.89, -1.31]	18.22
Singh 2016	20	174.80	22.44	20	195.50	32.29	-20.70 [-37.93, -3.47]	16.19
Gholaman 2018	10	257.30	8.16	10	259.40	14.84	-2.10 [-12.60, 8.40]	17.88
Kandhare 2018	48	206.47	36.16	53	198.01	39.52	8.46 [-6.37, 23.29]	16.85
Rashid 2019	32	176.70	19.33	32	228.51	9.67	-51.81 [-59.30, -44.32]	18.44
**Overall**							-16.50 [-34.38, 1.37]	

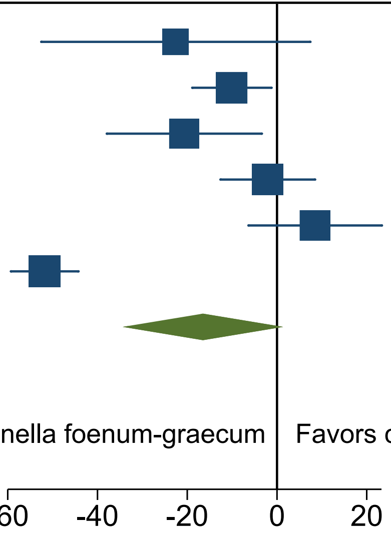

Heterogeneity: $\tau^2 = 436.51$, $I^2 = 92.46\%$, $H^2 = 13.27$

Test of $\theta_i = \theta_j$: Q(5) = 97.72, p = 0.00

Test of $\theta = 0$: z = -1.81, p = 0.07

Favors Trigonella foenum-graecum | Favors comparator

Random-effects REML model
